# Are maternal and child health initiatives helping to reduce under-five mortality in Ghana? Results of a quasi-experimental study using coarsened exact matching

**DOI:** 10.1186/s12887-021-02934-3

**Published:** 2021-10-25

**Authors:** Augusta Kolekang, Bismark Sarfo, Anthony Danso-Appiah, Duah Dwomoh, Patricia Akweongo

**Affiliations:** 1 University for Development Studies, Accra Tamale, Ghana; 2 School of Public Health, University of Ghana, Legon Accra, Ghana

**Keywords:** Child health, Under-five, Mortality, Interventions, Matching, And effectiveness

## Abstract

**Background:**

Despite a 53 % decline in under-five mortality (U5M) worldwide during the period of the Millennium Development Goals (MDGs), U5M remains a challenge. Under-five mortality decline in Ghana is slow and not parallel with the level of coverage of child health interventions. The interventions promoted to improve child survival include early initiation of breastfeeding, clean postnatal care, and skilled delivery. This study sought to assess the effectiveness of these interventions on U5M in Ghana.

**Methods:**

A quasi-experimental study was conducted using secondary data of the 2008 and 2014 Ghana Demographic and Health Surveys. Coarsened Exact Matching and logistic regression were done. The interventions assessed were iron intake, early initiation of breastfeeding, clean postnatal care, hygienic disposal of stool, antenatal care visits, skilled delivery, intermittent preventive treatment of malaria in pregnancy, and tetanus toxoid vaccine.

**Results:**

There were 2,045 children under-five years and 40 (1.9 %) deaths in 2008. In 2014, the total number of children under-five years was 4,053, while deaths were 53(1.2 %). In 2014, children less than one month old formed 1.6 % of all children under-five years, but 47.8 % of those who died. Mothers who attended four or more antenatal care visits were 78.2 % in 2008 and 87.0 % in 2014. Coverage levels of improved sanitation and water connection in the home were among the lowest, with 11.6 % for improved sanitation and 7.3 % for water connection in the home in 2014. Fifty-eight (58), 1.4 %, of children received all the eight (8) interventions in 2014, and none of those who received all these interventions died. After controlling for potential confounders, clean postnatal care was associated with a 66% reduction in the average odds of death (aOR = 0.34, 95 %CI:0.14–0.82), while early initiation of breastfeeding was associated with a 62 % reduction in the average odds of death (aOR = 0.38, 95 % CI: 0.21–0.69).

**Conclusions:**

Two (clean postnatal care and early initiation of breastfeeding) out of eight interventions were associated with a reduction in the average odds of death. Thus, a further decline in under-five mortality in Ghana will require an increase in the coverage levels of these two high-impact interventions.

**Supplementary Information:**

The online version contains supplementary material available at 10.1186/s12887-021-02934-3.

## Research in context

### What is already known?

- Increasing usage of child health interventions has been advocated to achieve a rapid decline in mortality. However, the effectiveness of these interventions can be sub-optimal, and some interventions are associated with an increased risk of death of children.

### What are the new findings?

- A quasi-experimental study design with coarsened exact matching was used to assess multiple interventions using secondary data.

### What do the new findings imply?

- Only a few interventions were associated with a reduction in mortality.
- To achieve a further decline in mortality, coverage of early initiation of breastfeeding and clean postnatal care should be increased since their coverage levels are low.
- Attention should be paid to children at higher risk of dying, including those with preceding birth interval less than two years, neonates, multiple births, and children from polygamous homes.

## Introduction

Under-five mortality (U5M) remains a major public health problem despite a 53 % decline globally between 1990 and 2015 [1]. Decline of U5M in Ghana is considered slow [2–4]. Thus, it could not achieve the Millennium Development Goal (MDG) 4 target [4, 5] despite several efforts at achieving it. According to Schieber, Cashin [2], Ghana’s under-five mortality rate is not commensurate with its high health care spending compared to other countries at the global level [2].

Variations in socio-demographic factors, including household, maternal, and child factors [6, 7], the prevalence of diseases and risk factors, and the impact of interventions [8–10] underpin the variations in mortality decline. To this end, maternal, neonatal, child health, and nutrition interventions have been advocated to reduce U5M. In Ghana, child health interventions include antenatal care visits, clean postnatal care, early initiation of breastfeeding, health facility delivery, skilled delivery, and household ownership of mosquito net. In addition, several strategies, including the Community-based Health Planning Services (CHPS) and the National Health Insurance Scheme (NHIS), have been implemented to increase access to and coverage of child health interventions and to improve survival [3]. These strategies resulted in increased coverage levels of most vaccines to 80 % in 2014 [11]. However, coverage levels of some other interventions, including clean postnatal care, improved sanitation, and water connection in the home, were below 30 % in Ghana in 2014 [3].

While low coverage of interventions leads to low impact [12–14], the impact of interventions can be low even when coverage is high if the quality of the interventions is poor or there are adverse events associated with the interventions. Poorer child survival has been documented with health facility and skilled delivery compared to home deliveries [15], iron intake [16], diphtheria, pertussis, and tetanus (DPT) vaccine [17, 18], and malaria vaccine [19, 20]. To achieve rapid mortality decline and possibly achieve the Sustainable Development Goals (SDG) 3 target 2, information on interventions with the potential to rapidly reduce mortality is required. This study, therefore, sought to assess the effectiveness of the child health interventions implemented in Ghana. The null hypothesis was that no intervention was associated with a decrease in U5M in Ghana during the period of the evaluation.

## Methods

### Study design

This was a quasi-experimental study design used to assess the effectiveness of eight [8] interventions. The Mosley and Chen framework for studying child survival in developing countries was adapted for this study [21]. The interventions were antenatal care visits, neonatal tetanus toxoid vaccine, clean postnatal care, hygienic disposal of stool, early initiation of breastfeeding, intermittent preventive treatment of malaria in pregnancy (IPT-p), iron intake, and skilled delivery. These are interventions expected to be implemented before, during, or shortly after birth, and that are therefore likely to have preceded (temporal association between exposure and outcome) death for those children who received them [22]. In addition, an overall or composite intervention group was created using children who received all eight [8] interventions. Definition of interventions can be found in the 2014 Ghana Demographic and Health Survey report [11]. For each child that received an intervention (treated), a control child who did not receive the intervention (untreated) was matched using coarsened exact matching [23–26].

### Study area

The study area was Ghana, a country in sub-Saharan Africa (SSA). There were ten [10] political regions and 216 districts in Ghana in 2008 and 2014. At the last census in 2010, the total population of Ghana was about 25 million [27]. The agriculture and services sectors contributed the most to the economy during the period of the evaluation [28]. On general health, the burden of illness or injury was 14 % in 2014, with individuals 50 years old and above (22.4 %) and children 0–5 years (20.3 %) reporting the highest burden [28].

Regarding the eating habits of Ghanaians, results from the literature are inconsistent. While Buxton [29] recorded that 63 % of Junior High Students in Ghana reported skipping breakfast on school-going days, Intiful and Lartey [30] recorded 86 % of students aged 6–19 years old in the Eastern Region of Ghana taking breakfast on the day they were interviewed. 45 % of adolescents reported eating two home-cooked meals a day, and 34 % preferred a soft drink during the day [29]. Rural dwellers consumed more starchy foods, while urban dwellers consumed more animal-based foods [31]. Skipping breakfast, low consumption of fruits and vegetables, and high consumption of energy-dense foods have also been reported among university students in Ghana [32].

### Study participants

The study population was children under-five years old in the Ghana Demographic and Health Surveys (GDHS) data sets born between 2003 and 2014. The analyses were restricted to usual household members and last births. The last birth restriction was done because interventions including antenatal care visits, iron intake, and clean postnatal care were measured for only the last births. It would also reduce the effect of recall bias on the study results. Only usual household members were also used because some interventions were not measured for non-usual household members.

### Data and source of data

Secondary data from the 2008 and 2014 GDHS were used. The data were collected by the Ghana Statistical Service (GSS). The GDHSs are complex household surveys, and the data were collected using a cross-sectional descriptive study design. Individuals were selected using a multistage cluster sampling technique [3]. The data are nationally representative, and response rates were over 90 % for both surveys used in this analysis [3]. Data sets of the 2008 and 2014 surveys were pooled for regression analysis. The years 2008 and 2014 were chosen because they have the data sets closest to the transition from the MDGs to the SDGs. Information from the study will therefore provide an understanding of the interventions that contributed to mortality reduction towards the end of the MDGs and, thus, should be focused on during the SDGs period to achieve rapid mortality reduction.

**Fig. 1:**
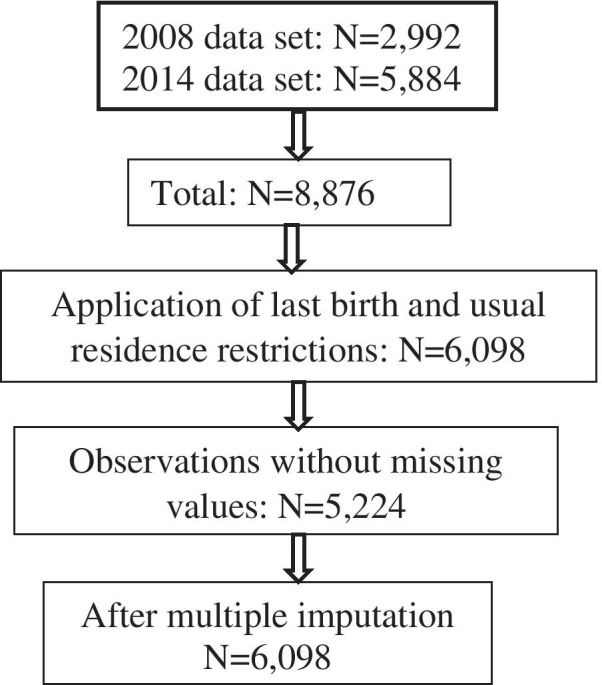
Flowchart of number of observations

The Household Recode (HR) and Kids Recode (KR) files were used for this analysis. The KR file was the main data set, and it contained information about children under-five years old at the start of the data collection whose mothers were interviewed [3]. The 2008 GDHS data set had 2,882 observations and 1,010 variables, while the 2014 had 5,884 observations and 1,159 variables (Fig. 1). The Stata versions of the data sets were downloaded from the DHS Program’s website (https://dhsprogram.com). The HR data sets were merged with the KR data sets so that household insecticide-treated nets/indoor residual spraying (ITN/IRS) information from the HR data set could be added to the KR data set. Household insecticide-treated nets/indoor residual spraying is an intervention and needed to be adjusted for in the analysis. The data sets of 2008 and 2014 were combined to obtain the final data set for the analysis.

48 % (48 %) of observations had at least one missing value for the covariates to be adjusted for, and therefore, multiple imputation was done before the regression analyses were done. Imputation was done to preserve the sample size and representativeness of the data. Variables with missing data were birth interval (16 observations), number of co-wives (832 observations), mode of delivery of child (2 observations), child’s weight (19 observations), employment status of mother (13 observations), NHIS status (1 observation), maternal BMI (2,601), anaemia (204), height (2,008) and child given other milk within three days after birth (58). The information on the missing data is presented in Additional file 3. Twenty (20) imputations were done using chain imputation and logistic regression. The imputation data format was marginal long, and the seed set was 200. Variables used in the multiple imputation were all variables without missing data that were used in the analysis. The design feature, weighting, was factored into the multiple imputation model. The Stata syntax of the multiple imputation is presented in Additional file 1.

### Study variables

The variables used in this analysis were socio-economic and demographic factors and interventions. Socio-economic factors were maternal and household variables, including maternal education, marital status, religion, ethnicity, household wealth, household size, number of children under-five years in a household, region, NHIS status, and place of residence. Demographic factors comprised child and maternal variables, including maternal age, child’s age and birth weight, birth interval, birth order, and multiple birth. Interventions were antenatal visit, IPT-p, iron intake, tetanus toxoid vaccine, skilled delivery, clean postnatal care, early initiation of breastfeeding, hygienic disposal of stool, water connection in the home, improved water source, improved sanitation, and ITN/IRS. The year of the survey was also included. The definition of variables is based on the 2014 GDHS report [3].

### Definition of interventions

Intermittent preventive treatment of malaria in pregnancy (IPT-p): Percent of pregnant women receiving 2 + doses of sulphadoxine-pyrimethamine/fansidar during pregnancy.

Iron intake: Percent of pregnant women taking an iron supplement daily for at least 90 days.

Tetanus toxoid vaccination: Percent of women who received two injections during the pregnancy of her last birth, or two or more injections (the last within 3 years of the last live birth), or three or more injections (the last within 5 years of the last birth), or four or more injections (the last within 10 years of the last live birth), or five or more injections at any time prior to the last birth.

Skilled delivery: Percent of children born with a skilled attendant present, including a medical doctor, nurse/midwife, and community health officer/nurse.

Clean postnatal care: Percent of neonates who received a preventive postnatal visit within 48 h of birth.

Early initiation of breastfeeding: Percent of children who started breastfeeding within 1 h of birth.

Hygienic disposal of stool: Percent of children’s stools that are disposed of safely. Stools are considered to be disposed of safely if: (1) the child always uses a toilet/latrine, (2) the feaces are thrown in the toilet/latrine, or (3) the feaces are buried in the yard.

Antenatal care visit: Percentage of women with a birth in the last 5 years receiving antenatal care from a skilled provider for the most recent birth.

### Pre-processing of data

Coarsened Exact Matching [23] was used to pre-process (match) the data to ensure balance in pre-treatment variables (covariates) between the treated and untreated groups. Covariate balance was assessed using linear 1 (L1) statistic. Linear 1 = 0 means perfect covariate balance between treated and untreated groups, while L1 = 1 means complete incompatible treatment and comparison groups with respect to the covariates. The closer the values of L1 to zero, the better the covariate balance [23]. Information on the sample size and covariate balance before and after matching are included in Additional file 2.

CEM puts observations into groups based on factors that influenced the decision to receive treatment. The groups were chosen by the first author to reflect clinically important categories and to be consistent with that in the GDHS reports. Observations in similar groups were matched, and treated and untreated individuals who did not have matches were deleted from the data set and the data analyzed as unmatched data. The covariates used in the matching process were variables that predicted intervention use from the literature review and were also associated with each intervention from the chi-square test done in this data analysis (Additional file 2).

### Analysis

Descriptive statistics were done and presented as numbers (percentages) by survival status (died or lived), and chi-squared test was used to assess the differences each for the years 2008 and 2014. Logistic regression was also fitted to assess factors associated with U5M after the multiple imputation using combined data of 2008 and 2014. The design features (stratification, clustering and weighting, and selection of primary sampling units) of the GDHS were factored into all the analyses. Also, the difference in women’s population between the two time periods was accounted for in the analysis. De-normalized weights were used to account for the differences in the population of women in the reproductive age in the population at the time of each survey. The de-normalized weight was calculated as = ψ X ϕ15–49/ϕ^S^15-49 [33]. Where ψ is the DHS sampling weight for women. ϕ15–49 is the population of women in the country at the time of each survey, while ϕ^S^15-49 is the total number of women 15–49 years interviewed during the data collection for each of the surveys as reported in the GDHS reports [11, 34].

Potential confounders adjusted for were those related to mortality and/or exposure from the conceptual framework/directed acyclic graphs (DAGs) (Additional file 4) and are not intermediates in the causal pathway of the exposure and outcome [35, 36]. Robust estimates of odds ratios (ORs) with 95 % confidence intervals were reported.

For the analysis of the effect of interventions on U5M, under the null hypothesis that no intervention had an effect on U5M, logistic regression was fitted using the pre-processed CEM data. Each intervention (antenatal care visit, IPT-p, iron intake, tetanus toxoid vaccine, skilled delivery, clean postnatal care, early initiation of breastfeeding, and hygienic disposal of stool) was the exposure, accounting for design features and controlling for potential confounders. Potential confounders adjusted for were those identified from the DAGs. Average treatment effects were reported as OR with 95 % CI and at a 2-tailed α level of 0.05 [37]. Analyses were performed using Stata version 13 [37]. The CEM syntax and algorithm are included in Additional file 1.

### Sensitivity analysis

#### Complete case analysis without maternal nutrition factors

When logistic regression was fitted with the completed cases, the total number of observations was 5,224, and early initiation of breastfeeding, child’s age, multiple birth, birth interval, number of children under-five years old in the household, and polygamy (co-wives) were associated with U5M from the adjusted analysis.

#### Multiple imputation with maternal nutrition factors

After multiple imputation, when maternal anaemia and Body Mass Index (BMI) were added, the sample size was reduced from 6,098 to 3,165 observations with very wide 95 % confidence intervals. The variable, water connection in the home, was omitted from the regression model. Body Mass Index and anaemia were not statistically significant, while household size, child’s age, multiple birth, maternal education, household indoor residual spraying and bed net ownership, and early initiation of breastfeeding were statistically significant. National Health Insurance Scheme status had borderline significance, p = 0.05. The confidence interval for multiple births ranged from 9.83 to 417.8.

#### Coarsened exact matching with completed cases without maternal nutrition factors

For the CEM analysis with completed cases, the sample size after matching for the regression analysis ranged from 4,914 to 6,043 observations. Only early initiation of breastfeeding was associated with U5M reduction from both the crude and adjusted analysis. Clean postnatal care and hygienic disposal of stool were significant from the crude analysis but not from the adjusted analysis.

## Results

### Descriptive statistics

There were 2,045 children under-five years and 40 (1.9 %) deaths in 2008. In 2014, the total number of children was 4,053, and deaths were 53 constituting 1.2 % (Table 1). In 2008, children less than one month constituted 2.2 % of all children under-five years, but 46.0 % among those who died. In 2014, children less than one month old formed 1.6 % of all children, but 47.8 % of those who died. About 78.2 % of mothers attended four or more antenatal care visits in 2008, increasing to 87.0 % in 2014 (Table 2). The interventions with the lowest coverage levels were improved sanitation and water connection in the home. The coverage level of improved sanitation was 7.8 % in 2008 and 11.6 % in 2014, while water connection in the home was 9.7 % in 2008 and 7.3 % in 2014. About 58(1.4 %) of children received all eight (8) interventions in 2014, and none of those who received all the eight interventions died.

**Table 1:** Background factors and mortality among children under-five years old in 2008 and 2014 (n = 6,098)

Year	Baseline (2008)				End line (2014)			
Characteristics	Total	Alive (98.1 %)	Died (1.9 %)	P-value	Total	Alive (98.8 %)	Died (1.2 %)	P-value
	n(%)	n(%)	n(%)		n(%)	n(%)	n(%)	
Age(months)								
< 1	46 (2.2)	26(1.4)	20 (46.0)	< 0.001	68 (1.6)	41(1.0)	27 (47.8)	< 0.001
1 to 5	294 (14.1)	288(14.1)	6 (13.5)		545 (12.7)	538(12.7)	7 (12.2)	
6 to 11	319 (15.2)	312(15.1)	7 (16.9)		577 (14.8)	569(14.7)	8 (15.2)	
12 to 59	1386 (68.6)	1379(69.4)	7 (23.6)		2863 (71.0)	2852(71.5)	11 (24.7)	
Sex								
Male	1043(51.5)	1016(51.2)	27(69.7)	0.03	2118(52.7)	2095(52.8)	23(43.1)	0.22
Female	1002 (48.4)	989(48.8)	13 (30.3)		1935 (47.3)	1905(47.2)	30 (56.9)	
Birth size)								
>=2.5 kg	1763(88.0)	1730(88.0)	33(86.8)	0.83	3545(88.5)	3503(88.5)	42(84.9)	0.45
< 2.5 kg	266 (12.0)	260(12.0)	6 (13.2)		505 (11.5)	495(11.5)	10 (15.1)	
Preceding birth interval								
>=2 years	1852(90.5)	1818(90.7)	34(81.5)	0.09	3700(90.8)	3654(90.9)	46(86.1)	0.37
< 2 years	188 (9.5)	182(9.3)	6 (18.5)		342 (9.2)	335(9.2)	7 (13.9)	
Birth order								
< 3	1191 (59.7)	1177(60.0)	14 (42.6)	0.04	2318 (59.8)	2295(59.9)	23 (44.6)	0.06
( > = 3)	854 (40.3)	828(40.0)	26 (57.4)		1735 (40.3)	1705(40.1)	30 (55.4)	
Gestation								
Singleton	1994 (97.5)	1959(97.7)	35 (85.7)	< 0.001	3951 (97.5)	3902(97.5)	49 (93.3)	0.05
Multiple birth	51 (2.5)	46(2.3)	5 (14.3)		102 (2.5)	98(2.5)	4 (6.7)	
Delivery type								
Vaginal	1913(92.9)	1877(93.1)	36(86.1)	NA	3611(86.8)	48(89.6)	3563(86.8)	0.65
Caesarean	130 (7.1)	126(7.0)	4 (13.9)		442 (10.5)	437(13.2)	5 (10.5)	
Had NHIS								
No	1198(60)	1174(59.9)	24(64.9)	0.56	1238(33.0)	1219(32.9)	19(39.4)	0.38
Yes	847(40)	831(40.1)	16(25.1)		2814(67.0)	2780(67.1)	34(60.6)	
Household size								
Less than 6	1099 (57.4)	1082(57.6)	17 (45.0)	0.14	2255 (59.1)	2230(59.3)	25 (46.1)	0.1
>=6	946 (42.6)	923(42.4)	23 (55.0)		1798 (40.9)	1770(40.7)	28 (53.9)	
Region								
Northern belt	676 (22.7)	657(22.6)	19 (33.8)	0.20	1363 (19.0)	1341(18.9)	22 (26.1)	0.21
Middle belt	669 (37.6)	657(37.6)	12 (38.8)		1221 (36.0)	1205(35.9)	16 (42.2)	
Southern belt	700 (39.6)	691(39.8)	9 (27.5)		1469 (45.0)	1454(45.2)	15 (31.7)	
Rural/urban residence								
Urban	725(40.3)	712(40.4)	13(35.2)	0.54	1669(46.2)	1651(46.3)	18(39.0)	0.38
Rural	1320 (59.7)	1293(59.6)	27 (64.8)		2384 (53.8)	2349(53.7)	35 (61.0)	
Wealth quintile								
Poorest	617 (23.2)	599(23.0)	18 (33.5)	0.67	1279 (21.7)	1258(21.5)	21 (33.0)	0.42
Poorer	450 (22.0)	443(22.1)	7 (19.6)		865 (20.1)	851(20.1)	14 (24.0)	
Middle	348 (18.6)	342(18.7)	6 (18.6)		750 (19.6)	742(19.6)	8 (16.2)	
Richer	368 (20.8)	363(20.8)	5 (18.7)		641 (19.6)	635(19.6)	6 (16.4)	
Richest	262 (15.4)	258(15.5)	4 (9.6)		518 (19.1)	514(19.2)	4 (10.5)	
Mother’s age								
< 35	1623(80.7)	1597(80.9)	26(72.0)	0.18	3148(78.0)	3110(78.1)	38(71.5)	0.35
35 to 49	422(19.3)	408(19.1)	14(28.0)		905(22.1)	890(21.9)	15(28.5)	
Maternal education								
None	748 (31.2)	730(31.1)	18 (37.1)	0.0001	1371 (26.6)	1347(26.5)	24 (36.7)	0.14
Primary	481 (24.2)	464(23.6)	17 (50.6)		824 (19.5)	814(19.4)	10 (25.9)	
Secondary or higher	816 (44.6)	811(45.3)	5 (12.3)		1858 (53.9)	1839(54.1)	19 (37.4)	
Religion								
Orthodox	530 (25.2)	522(25.3)	8 (19.7)	0.04	960 (21.7)	948(21.7)	12 (23.3)	0.54
Pentecostal	851 (46.2)	838(46.4)	13 (36.7)		1937 (54.5)	1917(54.7)	20 (44.3)	
Islam	399 (18.2)	390(18.2)	9 (18.7)		836 (16.8)	818(16.7)	18 (24.7)	
Others	265 (10.4)	255(10.1)	10 (24.9)		320 (7.0)	317(7.0)	3 (7.7)	
Ethnicity								
Akan	793 (46.5)	779(46.5)	14 (45.3)	0.98	1515 (46.5)	1502(46.7)	13 (33.7)	0.27
Mole-Dagbani	528 (20.4)	515(20.4)	13 (20.2)		1090 (17.3)	1074(17.3)	16 (20.9)	
Others(Ga/Dangbe/Eve)	724 (33.1)	711(33.1)	13 (34.5)		1448 (36.2)	1424(36.0)	24 (45.4)	
Married								
No	575(31.3)	566(31.4)	9(28.3)		1370(37.3)	1349(37.3)	21(44.6)	0.36
Yes	1470 (68.7)	1439(68.7)	31 (71.7)	0.72	2683 (62.7)	2651(62.7)	32 (55.5)	
Polygamous home								
No	1428(80.5)	1406(80.8)	22(68.2)		2801(84.8)	2773(84.9)	28(75.5)	0.1
Yes	386 (19.5)	374(19.2)	12 (31.8)	0.09	651 (15.2)	639(15.1)	12 (24.5)	
Mother’s employment status								
Not employed	248 (12.5)	241(12.3)	7 (19.5)	0.20	811(19.9)	800(19.9)	11(22.2)	0.72
Employed	1786 (87.5)	1753(87.7)	33 (80.5)		3240(80.1)	3198(80.1)	42(77.8)	
Number of children < 5 years in household								
1–2	1769 (86.9)	1736(87.0)	33 (82.3)	0.45	3557 (88.7)	3509(88.6)	48 (92.2)	0.39
3–9	276 (13.1)	269(13.0)	7 (17.7)		496 (11.3)	491(11.4)	5 (7.8)	
Maternal anaemia								
Not anaemic	788(38.9)	776(39.0)	12(34.2)	0.58	1169(56.7)	1153(56.6)	16(64.8)	0.45
Anaemic	1204(61.1)	1177(61.0)	27(65.8)		876(43.4)	867(43.4)	9(35.2)	
Maternal Body Mass Index (BMI)								
Normal	1120(63.2)	1110(63.3)	10(59.2)	0.77	1034(54.9)	1031(54.9)	3(35.9)	0.03
Abnormal	609(36.8)	603(36.8)	6(40.8)		734(45.2)	730(45.1)	4(64.2)	
Maternal height								
> 145 cm	1989(98.6)	1950(98.7)	39(96.9)	NA	2052(99.0)	2028(99.0)	24(97.4)	NA
<=145 cm	29(1.4)	28(1.3)	1(3.1)		20(1.0)	19(1.0)	1(2.6)	
Infant given other milk with 3days after birth								
No	1961(97.0)	1929(96.9)	32(100)	NA	3974(98.9)	3940(98.8)	34(100)	0.61
Yes	56(3.0)	56(3.0)	0(0.0)		49(1.2)	49(1.2)	0(0.0)	
Total	2045 (100)	2005(100)	40 (100)		4,053 (100)	4000(100)	53 (100)	

Birth interval, child’s weight, NHIS coverage, mother’s employment status, polygamous home, anaemia, maternal height, BMI, and child given other milk within three days after birth had missing data. Details of the missing data for 2008 and 2014 are presented in supplementary file 2

Source: 2008 and 2014 Ghana Demographic and Health Surveys

**Table 2:** Coverage of interventions and mortality among children under-five years old (n = 6,098)

Year	Baseline (2008)				End line (2014)			
Intervention	Total n (%)	Alive n(%)	Died n (%)	P-value	Total n (%)	Alive n(%)	Died n (%)	P-value
Water < 30 min	1600(77.5)	1566(77.5)	34(80.0)	0.72	2813 (64.2)	2772(64.1)	41 (72.7)	0.30
Water connected	175 (9.7)	173(9.9)	2 (17.0)	NA	294 (7.3)	288(7.2)	6 (11.8)	0.30
Improved sanitation	156 (7.8)	152(7.8)	4 (8.7)	NA	404 (11.6)	400(11.7)	4 (5.3)	0.10
Hygienic disposal of stool	918 (46.9)	913(47.6)	5 (11.2)	< 0.001	1474 (39.4)	1459(39.5)	15 (33.2)	0.45
Antenatal visit (4 plus)	1565 (78.2)	1541(78.4)	24 (68.4)	0.18	3480 (87.0)	3438(87.1)	42 (83.1)	0.4
IPT-p	830 (41.0)	812(40.9)	18 (46.2)	0.55	2828 (69.3)	2793(69.3)	35 (70.3)	0.90
Iron(90 day+)	781 (41.2)	765(41.1)	16 (46.4)	0.54	2266 (59.3)	2237(59.3)	29 (57.9)	0.86
TT protected	1416 (70.9)	1388(70.9)	28 (71.6)	0.93	3099 (79.0)	3063(79.1)	36 (69.1)	0.12
Health facility delivery	1153 (60.0)	1132(59.9)	21 (63.3)	0.71	2894 (74.7)	2861(74.8)	33 (64.7)	0.16
Skilled delivery	1183 (61.5)	1161(61.4)	22 (67.5)	0.48	2926 (75.4)	2893(75.6)	33 (64.7)	0.13
Contraceptives use	468 (23.3)	461(23.4)	7 (21.1)	0.77	1179 (29.9)	1169(28.0)	10 (21.6)	0.30
Clean postnatal	143 (6.4)	142(6.5)	1 (0.9)	NA	1010 (23.3)	1000(23.4)	10 (13.4)	0.07
Early breastfeeding	1,082 (51.7)	1071(52.3)	11 (24.6)	0.001	2245 (54.8)	2227(55.1)	18 (31.4)	0.002
Household ITN/IRS	1493 (71.1)	1467(71.5)	26 (59.1)	0.12	3491 (83.6)	3446(83.6)	45 (84.4)	0.90
8 interventions	0 (0.0)	0(0.0)	0 (0.0)	NA	58 (2.1)	58(2.1)	0 (0.0)	NA
Total	2045 (100)	2000 (100)	40 (100)		4053 (100)	4000 (100)	53 (100)	

N = total sample. n = number of observations. Eight interventions are antenatal visit, IPT-p, iron (90 day+), tetanus toxoid vaccine, skilled delivery, clean postnatal care, early initiation of breastfeeding, and household ITN/IRS coverage. *Statistical significance at 95 % confidence interval. Indoor Residual Spraying = ITN/IRS, Intermittent Preventive Treatment of Malaria in Pregnancy = IPT-p

Source: 2008 and 2014 Ghana Demographic and Health Surveys

## Interventions and under-five mortality

The crude and adjusted ORs of the effect of the interventions are presented in Tables 3 and 4. From the crude analysis, average odds of death was reduced by 68 % among those with early initiation of breastfeeding (OR = 0.32, 95 % CI: 0.19–0.54) and also 68 % among children under-five years who had clean postnatal care within 2 days (OR = 0.32, 95 %CI: 0.16–0.65). After adjusting for potential confounders, early initiation of breastfeeding reduced odds of death by 62 % (aOR = 0.38, 95 % CI: 0.21–0.69), while clean postnatal care was associated with a 66 % reduction in the average odds of death (aOR = 0.34, 95 %CI: 0.14–0.82) (Tables 3 and 4). Skilled delivery was not associated with U5M from the crude (OR = 1.10, 95 % CI: 0.55–2.20) and adjusted analysis (aOR = 1.77, 95 % CI: 0.60–5.25). Antenatal care visits was associated with U5M in the crude analysis (OR = 2.52, 95 % CI: 1.18–5.36) but not in the adjusted analysis (aOR = 1.59, 95 %CI: 0.71–3.57).

**Table 3:** Interventions associated with under-five mortality

Intervention	Sample size	OR(95 %CI)	aOR (95 %CI)
Early initiation of breastfeeding(within 1 h)	6,072	0.32(0.19–0.54)	0.38(0.21–0.69)
Clean postnatal care (within 2 days)	5,768	0.32(0.16–0.65)	0.34(0.14–0.82)
IPT-p	5,965	1.21(0.49–3.00)	1.64(0.76–3.54)
Skilled delivery	6,040	1.10(0.55–2.20)	1.77(0.60–5.25)
Tetanus toxoid vaccine	5,980	0.82(0.47–1.40)	0.86(0.41–1.79)
Hygienic disposal of stool	6,043	0.43(0.21–0.88)	0.74(0.33–1.66)
Iron intake(90days+)	6,045	1.43(0.77–2.67)	1.16(0.49–2.76)
Antenatal care (4 + visits)	5,951	2.52(1.18–5.36)	1.59(0.71–3.57)

Statistical significance with 95 % confidence interval, unadjusted odds ratios (OR), and adjusted odds ratios (aOR). Intermittent Preventive Treatment of Malaria in Pregnancy = IPT-p

Source: 2008 and 2014 Ghana Demographic and Health Surveys

**Table 4:** Adjusted odds ratios of interventions with effect on under-five mortality, multiple logistic regression analysis after matching

Covariates adjusted for in the model	Antenatal care visits	Skilled delivery	Iron intake	Early initiation of breastfeeding	Clean postnatal care	IPT-P	Hygienic disposal of stool	Tetanus toxoid vaccine
	aOR (95 %CI)	aOR (95 %CI)	aOR (95 %CI)	aOR (95 %CI)	aOR (95 %CI)	aOR (95 %CI)	aOR (95 %CI)	aOR (95 %CI)
Survey year								
2008								
2014	0.94(0.48–1.82)	1.18(0.58–2.39)	0.86(0.42–1.79)	0.99(0.52–1.89)	0.73(0.36–1.49)	10.74(0.79–3.82)	1.41(0.56–3.60)	0.99(0.53–1.85)
Age(months)								
< 1								
1 to 5	0.02(0.01–0.04)	0.01(0.003–0.02)	0.01(0.003–0.03)	0.02(0.01–0.06)	0.01(0.004–0.03)	0.005(0.001–0.02)	0.01(0.005–0.04)	0.02(0.01–0.04)
6 to 11	0.02(0.01–0.05)	0.01(0.003–0.03)	0.01(0.004–0.03)	0.02(0.01–0.06)	0.01(0.002–0.03)	0.01(0.002–0.03)	0.01(0.004–0.03)	0.02(0.01–0.05)
>=12 months	0.005(0.002–0.01)	0.003(0.001–0.01)	0.004(0.001–0.01)	0.01(0.003–0.02)	0.003(0.001–0.01)	0.002(0.001–0.01)	0.002(0.0005-0.01)	0.01(0.002–0.01)
Gender								
Male								
Female	0.86(0.46–1.62)	0.81(0.57–3.41)	0.84(0.38–1.86)	0.83(0.46–1.50)	0.85(0.40–1.81)	1.55(0.74–3.28)	1.04(0.47–2.32)	0.84(0.46–1.52)
Birth interval								
>=2 years								
Less than 2	1.92(0.85–4.38)	4.57(2.22–9.39)	1.38(0.64–2.95)	2.76(1.38–5.53)	2.91(1.43–5.88)	1.57(0.72–3.43)	1.49(0.61–3.61)	2.22(1.09–4.51)
Birth order								
Below 3								
>= 3	1.65(0.79–3.45)	2.41(0.98–5.90)	1.05(0.47–2.34)	1.85(0.88–3.89)	2.53(1.23–5.19)	1.93(0.87–4.27)	1.12(0.46–2.73)	1.60(0.76–3.34)
Birth weight								
Normal								
Small	0.86(0.33–2.20)	1.40(0.57–3.41)	NA	0.81(0.27–2.40)	0.69(0.26–1.83)	NA	0.74(0.21–2.54)	0.65(0.24–1.76)
Multiple birth								
No								
Yes	7.08(1.41–35.60)	1.08(0.15–7.94)	10.12(2.56-40.00)	2.56(0.32–20.27)	1.69(0.33–8.63)	3.617(0.83–15.63)	43.64(12.24-155.58)	7.48(1.81–30.98)
Delivery type								
Vaginal								
Caesarean	0.89(0.36–2.21)	0.67(0.28–1.59)	0.66(0.26–1.69)	0.89(0.35–2.27)	0.52(0.19–1.43)	0.38(0.13–1.08)	0.48(0.16–1.46)	0.83(0.32–2.14)
Region								
Northern belt								
Middle belt	0.80(0.33–1.94)	0.61(0.23–1.56)	1.20(0.46–3.18)	0.59(0.25–1.39)	0.90(0.29–2.75)	0.62(0.21–1.82)	0.91(0.32–2.58)	0.75(0.31–1.80)
Southern belt	0.36(0.12–1.09)	0.42(0.13–1.39)	0.63(0.18–2.19)	0.45(0.16–1.28)	0.81(0.22–3.06)	0.28(0.07–1.10)	0.31(0.07–1.32)	0.39(0.14–1.10)
Urban/rural residence								
Urban								
Rural	1.16(0.45–3.01)	1.40(0.44–4.44)	0.92(0.35–2.42)	0.76(0.30–1.93)	0.83(0.32–2.12)	1.61(0.51–5.11)	2.02(0.52–7.81)	1.09(0.39–3.05)
Wealth quintile								
Poorest								
Poorer	0.85(0.28–2.57)	0.59(0.18–1.96)	0.92(0.28–3.04)	0.98(0.36–2.65)	1.52(0.37–6.29)	0.91(0.28-3.00)	0.62(0.21–1.83)	0.85(0.31–2.32)
Middle	1.32(0.41–4.23)	1.57(0.45–5.49)	1.20(0.33–4.33)	1.15(0.32–4.09)	2.17(0.49–9.68)	4.67(1.14–19.17)	1.35(0.28–6.54)	1.18(0.37–3.79)
Richer	0.79(0.16-4.00)	1.47(0.26–8.48)	1.06(0.22–5.18)	0.86(0.18–4.03)	0.88(0.11–7.07)	1.09(0.16–7.67)	0.79(0.14–4.63)	1.03(0.20–5.36)
Richest	1.35(0.23–7.73)	2.62(0.40-17.16)	1.24(0.23–6.75)	1.75(0.30-10.08)	3.85(0.34–43.59)	3.61(0.42–22.21)	1.15(0.15–8.76)	1.64(0.25–10.60)
Polygamous home								
Not polygamous								
Polygamous	2.22(1.15–4.28)	2.14(1.01–4.51)	1.64(0.73–3.70)	2.82(1.53–5.17)	3.16(1.44–6.95)	1.52(0.70–3.33)	0.94(0.29–3.05)	1.95(1.06–3.60)
Household size								
< 6								
>=6	1.57(0.72–3.41)	1.49(0.68–3.27)	2.12(0.96–4.68)	1.22(0.56–2.68)	1.60(0.77–3.36)	2.47(1.12–5.43)	1.31(0.60–2.84)	1.24(0.58–2.67)
No. of CU5 in household								
1–2								
3 and above	0.18(0.06–0.53)	0.12(0.03–0.44)	0.16(0.05–0.51)	0.15(0.05–0.50)	0.07(0.02–0.23)	0.17(0.06–0.44)	0.15(0.04–0.55)	0.20(0.07–0.54)
Maternal age								
< 35								
>=35	0.67(0.30–1.51)	0.60(0.24–1.48)	1.03(0.46–2.33)	0.57(0.24–1.32)	0.54(0.21–1.35)	0.39(0.18–0.84)	0.34(0.11-1.00)	0.73(0.35–1.55)
Maternal education								
None								
Primary	1.38(0.54–3.52)	1.45(0.52–4.05)	2.13(0.75–6.05)	1.52(0.59–3.93)	1.39(0.58–3.32)	0.72(0.27–1.88)	1.33(0.52–3.39)	1.06(0.43–2.66)
Secondary or higher	0.59(0.21–1.65)	0.83(0.28–2.46)	0.71(0.26–1.91)	0.57(0.21–1.59)	0.84(0.34–2.10)	0.34(0.12–0.98)	0.63(0.19–2.05)	0.49(0.19–1.28)
Married								
Not Married								
Married	0.70(0.32–1.53)	0.38(0.17–0.87)	0.73(0.29–1.82)	0.64(0.31–1.35)	0.67(0.31–1.46)	0.71(0.32–1.59)	0.71(0.27–1.86)	0.71(0.34–1.47)
Religion								
Orthodox								
Pentecostal	0.85(0.37–1.95)	1.41(0.52–3.79)	0.61(0.22–1.67)	1.13(0.49–2.61)	0.94(0.38–2.33)	0.72(0.27–1.95)	0.58(0.22–1.54)	0.81(0.37–1.78)
Islam	1.30(0.48–3.51)	3.37(1.06–10.73)	1.22(0.36–4.13)	2.13(0.79–5.77)	3.81(1.27–11.39)	2.59(0.95–7.10)	0.61(0.18–2.09)	1.35(0.54–3.41)
Others	1.17(0.37–3.70)	4.14(0.94–18.20)	0.93(0.19–4.45)	2.17(0.67–7.06)	1.90(0.52–6.98)	2.66(0.77–9.25)	0.98(0.30–3.16)	1.31(0.41–4.15)
Ethnicity								
Akan								
Mole-Dagbani	0.56(0.15–2.01)	1.01(0.27–3.85)	0.79(0.23–2.75)	0.48(0.15–1.52)	0.53(0.15–1.91)	0.50(0.12–2.07)	1.29(0.38–4.42)	0.65(0.20–2.05)
Others	1.26(0.54–2.95)	1.20(0.52–2.78)	1.53(0.53–4.37)	0.86(0.40–1.86)	1.78(0.77–4.12)	1.03(0.41–2.54)	1.22(0.48–3.08)	1.30(0.58–2.93)
Mother’s employment								
Not employed								
Employed	0.67(0.29–1.54)	0.46(0.21–1.01)	0.64(0.27–1.51)	0.90(0.40–2.04)	1.41(0.53–3.73)	0.84(0.36–1.97)	0.77(0.28–2.11)	0.74(0.34–1.65)
Contraceptives use								
No use								
Use	1.19(0.51–2.78)	1.36(0.54–3.44)	0.89(0.36–2.23)	1.40(0.62–3.14)	1.52(0.62–3.72)	0.90(0.41–1.97)	1.07(0.29–3.88)	1.43(0.64–3.16)
Sanitation								
Not improved								
Improved	1.05(0.40–2.74)	1.05(0.37–3.02)	0.60(0.21–1.72)	0.95(0.23–3.98)	0.48(0.12–1.88)	0.65(0.19–2.15)	0.88(0.30–2.58)	0.90(0.33–2.42)
Antenatal visits								
< 4plus visits								
4 + visit	1.59(0.71–3.57)	1.22(0.47–3.15)	0.88(0.26–2.96)	0.84(0.41–1.72)	0.91(0.37–2.24)	2.05(0.69–6.10)	0.99(0.39–2.50)	0.88(0.40–1.91)
Tetanus toxoid vaccine								
Not received								
Received	0.85(0.37–1.94)	0.59(0.26–1.33)	1.53(0.57–4.11)	0.57(0.26–1.25)	0.49(0.23–1.05)	0.58(0.23–1.35)	2.06(0.91–4.69)	0.86(0.41–1.79)
Skilled delivery								
Not skilled								
Skilled	1.48(0.54–4.03)	1.77(0.60–5.25)	1.71(0.61–4.77)	1.58(0.61–4.11)	1.58(0.51–4.91)	2.01(0.69–5.83)	0.94(0.32–2.80)	1.70(0.68–4.24)
Improved water source								
Not improved								
Improved	1.40(0.61–3.21)	1.39(0.56–3.42)	2.66(0.86–8.23)	1.34(0.63–2.82)	1.33(0.56–3.16)	1.76(0.69–4.65)	0.98(0.39–2.46)	1.31(0.61–2.82)
Water connected								
Not connected								
Connected	0.77(0.21–2.78)	0.48(0.13–1.88)	0.58(0.11–2.94)	0.52(0.14–1.96)	0.84(0.18–3.85)	0.46(0.08–2.72)	0.42(0.09–1.95)	0.64(0.17–2.38)
ITN/IRS protection								
Not protected								
Protected	0.83(0.37–1.87)	0.86(0.32–2.35)	1.02(0.42–2.50)	0.79(0.37–1.68)	0.76(0.38–1.53)	1.25(0.41–3.75)	0.37(0.16–0.87)	0.79(0.37–1.72)
Hygienic disposal of stool								
Not hygienic								
Hygienic	0.54(0.28–1.05)	0.63(0.30–1.32)	0.56(0.26–1.18)	0.58(0.31–1.06)	0.52(0.24–1.11)	0.43(0.20–0.91)	0.74(0.33–1.66)	0.62(0.33–1.15)
Early breastfeeding								
Not early initiation								
Early initiation	0.37(0.19–0.73)	0.32(0.14–0.72)	0.41(0.17–0.99)	0.38(0.21–0.69)	0.57(0.28–1.18)	0.21(0.10–0.46)	0.29(0.11–0.77)	0.42(0.22–0.79)
Clean postnatal care in 2days								
No clean postnatal								
Clean postnatal	0.29(0.11–0.75)	0.20(0.05–0.76)	0.24(0.06–1.03)	0.43(0.19–0.97)	0.34(0.14–0.82)	0.15(0.05–0.46)	0.27(0.05–1.46)	0.40(0.16–1.01)
Iron intake > 90 days								
No iron intake								
Iron intake	1.51(0.73–3.11)	1.30(0.62–2.72)	1.16(0.49–2.76)	1.31(0.64–2.65)	1.33(0.58–3.09)	1.62(0.79–3.30)	2.26(0.83–6.17)	1.51(0.73–3.10)
IPT-p								
No IPT-p								
IPT-p	1.65(0.76–3.57)	2.36(1.14–4.89)	1.74(0.78–3.92)	1.74(0.89–3.39)	1.20(0.65–2.22)	1.74(0.79–3.82)	0.81(0.35–1.86)	1.47(0.74–2.94)
NHIS status								
Not covered								
Covered	0.50(0.27–0.91)	0.51(0.27–0.98)	0.68(0.33–1.38)	0.68(0.38–1.22)	0.33(0.14–0.75)	0.55(0.25–1.22)	0.74(0.34–1.61)	0.51(0.28–0.94)

NB: Not applicable. The factor is an intermediate in the causal pathway and was not adjusted for in the model. Statistical significance with 95 % confidence interval, unadjusted odds ratios (OR), and adjusted odds ratios (aOR)

## Socio-demographic factors and interventions associated with under-five mortality

Results of the association of child, maternal and socio-demographic factors with U5M are presented in Table 5. The crude and adjusted ORs and 95 % CI for the associations are presented. On child factors, after controlling for potential confounders, compared to children less than 1month old, odds of death was reduced by 98 % (aOR = 0.02, 95 %CI: 0.01–0.04) among children 1–5 months. Also, a 98 % reduction of odds of death was reported among those 6–11 months (aOR = 0.02, 95 % CI: 0.01–0.05) and 99 % among those 12–59 months (aOR = 0.01, 95 %CI: 0.002–0.01) relative to those less than 1 month old. Children who were multiple births were 7 times more likely to die compared to singleton births (aOR = 6.55, 95 % CI: 1.56–27.57), while those with preceding birth intervals of less than two years twice more likely to die relative to those with longer birth interval (aOR = 2.17, 95 %CI: 1.07–4.40). On household factors, children in households with 3 or more children under-five years had their odds of death reduced by 81 % (aOR = 0.19, 95 % CI: 0.07–0.54) compared to those in households with one or two children under five years old. Also, children in polygamous homes were 2 times more likely to die compared to children in monogamous homes (aOR = 1.94, 95 % CI: 1.05–3.61). The odds of death was reduced by 45 % among children whose mothers had health insurance coverage (aOR = 0.55, 95 % CI: 0.31–0.96).

**Table 5:** Factors associated with under-five mortality among children 0–59 months without matching (N = 6,098)

Characteristics	OR (95 % CI)	aOR(95 % CI)
Survey year		
2008		
2014	0.63(0.40-1.0)	0.89(0.49–1.62)
Age(months)		
< 1		
1 to 5	0.02(0.01–0.05)	0.02(0.01–0.04)
6 to 11	0.03(0.01–0.06)	0.02(0.01–0.05)
12 and above	0.01(0.004–0.02	0.01(0.002–0.01)
Gender		
Male		
Female	0.90(0.57–1.41)	0.87(0.49–1.56)
Birth interval		
>=2 years		
Less than 2	1.88(0.94–3.76)	2.17(1.07–4.40)
Birth order		
Below 3		
>= 3	1.95(1.21–3.14)	1.80(0.87–3.68)
Birth weight		
Normal		
Small	1.26(0.67- 2.37)	0.84(0.32–2.24)
Multiple birth		
Singleton		
Multiple birth	4.99(2.20-11.33)	6.55(1.56–27.57)
Delivery type		
Vaginal		
Caesarean	1.09(0.50–2.41)	0.87(0.35–2.17)
Region		
Northern belt		
Middle belt	0.74(0.43–1.28)	0.75(0.32–1.75)
Southern belt	0.46(0.26–0.83)	0.44(0.16–1.23)
Urban/rural residence		
Urban		
Rural	1.34(0.82–2.18)	0.86(0.35–2.16)
Wealth quintile		
Poorest		
Poorer	0.70(0.37–1.33)	0.86(0.32–2.32)
Middle	0.59(0.29–1.19)	1.00(0.32–3.18)
Richer	0.58(0.27–1.22)	0.82(0.19–3.57)
Richest	0.37(0.15–0.94)	1.21(0.24–6.21)
Polygamous home		
Not polygamous		
Polygamous	1.99(1.14–3.49)	1.94(1.05–3.61)
Household size		
< 6		
>=6	1.69(1.05–2.72)	1.01(0.51–2.36)
No. of CU5 in household		
1–2		
3 and above	1.03(0.52–2.04)	0.19(0.07–0.54)
Maternal age		
< 35		
>=35	1.19(0.73–1.93)	0.81(0.38–1.71)
Maternal education		
None		
Primary	1.44(0.82–2.53)	1.16(0.48–2.80)
Secondary or higher	0.36(0.20–0.65)	0.51(0.20–1.30)
Married		
Not Married		
Married	0.92(0.56–1.50)	0.69(0.34–1.39)
Religion		
Orthodox		
Pentecostal	0.83(0.45–1.55)	0.90(0.40–2.01)
Islam	1.35(0.67–2.72)	1.32(0.52–3.34)
Others	2.02(0.90–4.55)	1.26(0.41–3.86)
Ethnicity		
Akan		
Mole-Dagbani	1.35(0.74–2.45)	0.66(0.21–2.08)
Others	1.39(0.80–2.40)	1.25(0.57–2.73)
Mother’s employment		
Not employed		
Employed	0.76(0.42–1.37)	0.76(0.34–1.72)
Contraceptives use		
No use		
Use	0.71(0.38–1.30)	1.40(0.63–3.08)
Sanitation		
Not improved		
Improved	0.64(0.29–1.38)	0.91(0.35–2.36)
Antenatal visits		
< 4plus visits		
4 + visit	0.61(0.36–1.04)	0.82(0.40–1.69)
Tetanus toxoid vaccine		
Not received		
Received	0.73(0.44–1.21)	0.83(0.40–1.75)
Skilled delivery		
Not skilled		
Skilled	0.80(0.49–1.32)	1.57(0.64–3.89)
Improved water source		
Not improved		
Improved	1.45(0.82–2.55)	1.40(0.66–2.98)
Water connected		
Not connected		
Connected	0.89(0.35–2.26)	0.65(0.19–2.24)
ITN/IRS protection		
Not protected		
Protected	0.70(0.41–1.20)	0.77(0.37–1.60)
Hygienic disposal of stool		
Not hygienic		
Hygienic	0.35(0.20–0.62)	0.59(0.32–1.07)
Clean postnatal care in 2days		
No clean postnatal		
Clean postnatal	0.36(0.18–0.71)	0.40(0.16–0.99)
Iron intake > 90 days		
No iron intake		
Iron intake	1.01(0.63–1.61)	1.49(0.75–2.94)
Early breastfeeding		
Not early		
Early	0.33(0.2–0.54)	0.39(0.21–0.72)
IPT-p		
No IPT-p		
IPT-p	1.00(0.62–1.63)	1.47(0.76–2.86)
NHIS status		
Not covered		
Covered	0.70(0.44–1.13)	0.55(0.31–0.96)

Statistical significance with 95 % confidence interval, unadjusted odds ratios (OR), and adjusted odds ratios (aOR)

## Discussion

### Interventions and under-five mortality

Despite efforts at reducing U5M, mortality decline in Ghana is slow. Although interventions targeted at reducing under-five mortality might show efficacy under experimental conditions, their effectiveness might be suboptimal, resulting in their low impact on mortality. This study evaluated the effectiveness of the various child health interventions on U5M in Ghana. The results showed that the various child health interventions impact child mortality differently. Considering that increase in coverage of interventions is the main strategy to address U5M and possibly achieve the SDG 3 target 2, the lack of effect of key interventions on U5M implies that an increase in coverage alone might not yield the needed decline in mortality to achieve the SDG target.

Children who had clean postnatal care had a 66 % reduced average odds of death. Clean postnatal care is defined as neonates receiving a preventive postnatal visit within 48 h of delivery [3]. The assumption is that neonates who receive clean postnatal care will subsequently receive adequate clean postnatal care in the home [3, 38]. Sepsis is a major cause of neonatal mortality, and cord care influences the incidence of neonatal sepsis. Therefore, clean birth and postnatal care practices are recommended for reducing infection and neonatal mortality [39], and thus, the recommendation that women after delivery have postnatal care within 48 h. This will ensure that infections are identified early for timely management.

Clean postnatal care can significantly contribute to the elimination of neonatal tetanus which is a significant contributor to neonatal mortality[40]. From the 2014 Ghana Demographic and Health Survey, mothers received clean postnatal care from skilled or traditional birth attendants. Skilled health workers include doctors, nurses, midwives, community health nurses, or community health officers. Clean postnatal care is one of the interventions with the lowest coverage levels in Ghana. Coverage in 2008 was 6.5 %, while that in 2014 was 22.8 % [11]. Its low coverage is, therefore, an avenue to further reduce deaths if its coverage level is increased. The result of the protective effect of clean postnatal care on mortality in this study is similar to that documented [5, 41–43].

Breastfeeding has been shown to reduce the risk of infections and, consequently, the death of children under-five years old [41, 44–47]. Early breastfeeding has additional benefits as it promotes warmth and bonding between infants and their mothers. In this study, early initiation of breastfeeding was associated with a 62 % reduction in the average odds of death. Similar results of the association between early initiation of breastfeeding and under-five mortality have been documented [44–48]. According to the GDHS, the coverage level of early initiation of breastfeeding was 25.5 % in 1998, 46.3 % in 2003, 52.3 % in 2008, and 55.6 % in 2014 [11, 38]. This trend of coverage increase is slow. Considering the 2014 coverage level of the intervention, it has the potential to further reduced mortality if its coverage level is increased. Since this intervention does not require much logistics or expenditure to implement compared to interventions like skilled delivery, it should be prioritized.

The lack of effect of the other interventions on under-five mortality could be attributed to the context of the implementation of these interventions. The context could include the incidence of the disease(s) each intervention is targeted at and the quality of the implementation of the interventions. There is evidence of poor quality of skilled delivery contributing to lack of effect of skilled delivery on child mortality [49]. Disparities in the association of skilled delivery with neonatal mortality have been documented in different geographic areas. While skilled delivery improved neonatal mortality in Latin America and the Caribbean, it was associated with worse neonatal mortality in Africa [50]. The quality and availability of logistics and health personnel for skilled delivery might explain the differences in the effectiveness of skilled delivery in different places [50].

Low incidence of disease that the intervention is targeted at could also explain the lack of statistical significance of the association between interventions and under-five mortality [51]. With a low incidence of disease, the intervention will have fewer deaths to prevent and, therefore, low effectiveness. That could be the case for the tetanus toxoid vaccine. In addition, improvement in birthing practices could reduce infections due to *Clostridium tetani*, and therefore, low risk of neonatal tetanus, a resultant reduced risk of death from neonatal tetanus. Currently, Ghana is at the elimination state of neonatal tetanus, which means a lower risk of neonatal tetanus infection [51].

With multiple interventions which have a direct and indirect effect on under-five mortality been implemented in the midst of socio-economic factors that also affect under-five mortality, Chowdhury [52] observed that proximate factors have a stronger effect on under-five mortality than more distal factors. Iron intake, antenatal care visits, and intermittent preventive treatment of malaria in pregnancy (IPT-p) have a relatively indirect effect on U5M and, therefore, could account for their lack of statistically significant effect on mortality.

### Other factors and under-five mortality

In the midst of interventions, some socio-demographic factors remained associated with U5M. Children in households with three or more children under–five years were 81 % less likely to die than those with one or two children. Having more children under-five years could mean the mother will have more experience taking care of children, including identifying signs and symptoms of diseases. More children under-five years in a household have been associated with early care-seeking in Niger [53]. Children in households with a higher number of members also had reduced odds of neonatal and under-five mortality in Ghana [33].

Health insurance membership offers financial access to healthcare and is associated with increased and timely healthcare-seeking [54–56]. It could therefore increase the use of all the healthcare-associated interventions, and thus, its association with a 45 % reduction in the odds of death of children under-five years in this study. A similar result of the protective effect of NHIS on mortality has been reported [33].

The higher odds of death of younger children and multiple births have been documented [41]. In this study, while multiple births were about 7 times more likely to die, compared to neonates, odds of deaths was reduced by about 99% among children 12 months and older. Addressing the increased odds of death with multiple births and younger children will require improved access to quality skilled delivery. Unfortunately, skilled delivery was not associated with mortality reduction. Younger children, especially neonates, are less developed and more susceptible to infections. However, specialized and advanced care needed during the neonatal period is unavailable, especially in poverty-ridden communities. Considering the causes of neonatal deaths such as sepsis, diarrhoea, pneumonia, and asphyxia, quality skilled delivery, improved nutrition, and improved hygiene will play essential roles in its reduction [57]. But, coverages of hygiene and sanitation interventions are among the interventions with the lowest coverage levels in Ghana [3, 38]. Also, coverage levels of exclusive breastfeeding and complementary feeding are on the decline [3, 38].

Additionally, children from mothers in polygamous marriages had twice higher odds of death. This finding is consistent with results reported in other studies [58, 59]. Rivalry among wives was attributed to the higher odds of death. Limited resources and overcrowding have also been cited as reasons for the positive correlation of polygamy and child mortality [60].

Lastly, children of birth intervals of less than two years also had twice higher odds of death compared to those of longer intervals. With shorter birth intervals, mothers might not have replenished nutrients used during the previous pregnancy resulting in under-nutrition [61]. After delivery, there will be competition for maternal resources, including time to care for the children. This can compromise the quality of their care resulting in infections, improper nutrition, and poor health. A shorter birth interval will also limit the duration of breastfeeding, which can affect the development of the older child. Similar results on the higher risk of death among children with shorter birth intervals have been documented in Ghana [5, 62].

### Limitations and strength of the study

On limitations, exact matching coarsened like other matching methods matches on measured variables (potential confounders) contained in the data set, unlike randomization that controls for measured and unmeasured potential confounders. However, balanced was achieved between the treated and untreated groups using the variables available in the data set. Additionally, potential confounders adjusted for in this multiple regression analysis comprised all measured potential confounders, and therefore, the effect of unobserved variables is ignorable.

Also, 48 % of observations had at least one missing value. Multiple imputation was done to address the missing data. On the strengths of the study, nationally representative data were used, which had high response rates (over 90 %), and therefore, the study findings are generalizable. The pooling of the data also increased the power of the study and, thus, the validity of the results. The matching method that was used reduced model dependence and thus, increases the robustness of the analysis. Lastly, the restriction of the analysis to only the last births reduced the influence of recall bias on the study results.

## Conclusions

Of the eight (8) interventions assessed for effectiveness at reducing under-five mortality, two (2) showed effectiveness on mortality reduction. This, therefore, suggests that the scope and content of the current package of interventions targeted at reducing U5M will likely not achieve a rapid decline in mortality. At best, the mortality rate will be stagnant. To achieve a further decline in mortality, coverage of early initiation of breastfeeding and clean postnatal care should be increased. Further work should be done to understand the lack of association between some interventions, especially skilled delivery and U5M. Lastly, addressing issues affecting the health of children in polygamous homes and multiple births and improving National Health Insurance Scheme coverage could be beneficial.

## Supplementary Information

**Additional file 1.**

**Additional file 2.**

**Additional file 3.**

**Additional file 4.**

## Data Availability

The datasets used and/or analyzed during the current study are available from the corresponding author on reasonable request.

## Abbreviations

CEM: Coarsened Exact Matching
CHPS: Community-based Health Planning Services
DAGs: Directed Acyclic Graphs
DPT: Diphtheria, Pertussis, and Tetanus
GDHS: Ghana Demographic and Health Survey
GHS: Ghana Health Service
HR: Household Recode
ITN/IRS: Household Insecticide Treated Bed Nets and Indoor Residual Spraying
IPT-P: Intermittent Preventive Treatment of Malaria in Pregnancy
KR: Kids Recode
MDGs: Millennium Development Goals
MOH: Ministry of Health
NHIS: National Health Insurance Scheme
OR: Odds Ratio
SDGs: Sustainable Development Goals
SSA: Sub-Saharan Africa
U5M: Under-five Mortality
WHO: World Health Organization
