# Supplementary material for: Are maternal and child health initiatives helping to reduce under-five mortality in Ghana? Results of a quasi-experimental study using coarsened exact matching
Source: BMC Pediatr. 2021 Oct 25;21:473. doi: 10.1186/s12887-021-02934-3 (PMC8547109; doi:10.1186/s12887-021-02934-3)
Supplement: Supplementary file 1 — Additional file 1. [file 12887_2021_2934_MOESM1_ESM.docx]

**Methods appendix: Coarsened exact matching (CEM) algorithm and stata syntax**

**CEM algorithm**

Step 1: To match members across the control and intervention groups, a representational set of properties were chosen to identify the different archetypes of study participants.

Step 2: The CEM algorithm was then designed in such a way that members in the control and intervention groups fall into comparable archetypes (bin signatures)

Step 3: Every member of the target population was represented by properties coarsened to discrete values using a coarsening or binning strategy. Thus, each individual is given a “BIN Signature” that was used to exactly match other members with the same “BIN Signature”. The BIN signature development was based on the following variables: survey year, region, religion, ethnicity, maternal education, birth order, national health insurance status, multiple births, skilled delivery, polygamous home, antenatal care visits, urban or rural residence and type of delivery.

Step 4: With every member having a BIN Signature, each member in the control group is matched to other members of the intervention group with that same BIN Signature.

Step 5: The algorithm takes the difference of the outcome measure between the matched control and the corresponding intervention individual.

Step 6: Finally, the individual level impact is averaged across the entire sample.

**Stata syntax**

set more off

lookfor _merge

ta _merge

ta _merge, nol

gen new_weight=.

replace new_weight=wgt*83583/4916 if v007==2008

replace new_weight=wgt*102211/9396 if v007==2014

svyset [pw=new_weight], psu(v021) strata(v022)

ta region /*n=8876*/

***EXCLUSION CRITERIA**

ta midx

tab midx, m

drop if midx>1 /*restriction to last births*/

drop if v116==97 /*restricted analysis to usual residence*/

drop if v137==0 /*restricted to households with children under-five years*/

ta region /*n=6098*/

**Multiple imputation model**

mi set mlong

mi misstable summarize cowives birthinter childweigt delity NHIStatus workingcg

mi register imputed cowives birthinter childweigt delity NHIStatus workingcg

mi impute chained ///

(logit, aug) cowives ///

(logit, aug) birthinter ///

(logit, aug) childweigt ///

(logit, aug) delity ///

(logit, aug) NHIStatus ///

(logit, aug) workingcg ///

=died sex birthorder timevtCAT materage3 matereduc ///

maritstatus2 contracept hhmem hhchu5 sexhhd regioncem ruralurban wealth ///

ttprotect improsani ITNIRS skideli postnatal ANCVISITS ///

surveyyear IPTp hygincdis religion2 ethnicity ///

imprdrkgwat irondays multibirth earlybreastfd watercont ///

[pw=new_weight], add(20) rseed(200)

*LOGISTIC

mi estimate, esampvaryok or: svy:logistic died ///

surveyyear sex childweigt i.birthinter birthorder ///

multibirth i.timevtCAT materage3 i.matereduc i.maritstatus2 hhmem ///

i.regioncem ruralurban i.wealth workingcg delity i.religion2 i.ethnicity ///

cowives IPTp irondays ttprotect ///

skideli earlybreastfd postnatal ANCVISITS improsani ITNIRS imprdrkgwat hygincdis ///

watercont contracept NHIS hhchu5

***CEM analysis**

*CEM analysis

*Tetanus

imb surveyyear wealth ruralurban matereduc NHIStatus ANCVISITS ,tr(ttprotect)

cem surveyyear(#0) wealth(#0) matereduc(#0) ruralurban(#0) ///

NHIStatus(#0) ANCVISITS(#0) ,tr(ttprotect)

drop if cem_match==0

gen newcemw=new_weight*cem_weights

mi estimate, esampvaryok or:svy:logistic died ttprotect

mi estimate, esampvaryok or:svy:logistic died ttprotect sex childweigt i.birthinter birthorder ///

multibirth i.timevtCAT materage3 i.matereduc i.maritstatus2 contracept ///

i.regioncem ruralurban i.wealth improsani ITNIRS skideli postnatal ANCVISITS ///

/*ttprotect*/ IPTp irondays surveyyear imprdrkgwat hygincdis delity i.religion2 ///

i.ethnicity cowives hhmem workingcg earlybreastfd watercont NHIStatus hhchu5

*IRON INTAKE

imb surveyyear regioncem wealth birthinter ANCVISITS,tr(irondays)

L=0.36

cem surveyyear(#0) regioncem(#0) wealth(#0) ///

birthinter(#0) ANCVISITS(#0) ,tr(irondays) misets(20) sh

drop if cem_match==0

gen newcemw=new_weight*cem_weights

mi svyset [iweight=newcemw], psu(v021) strata(v022)

mi estimate, esampvaryok or:svy:logistic died irondays

mi estimate, esampvaryok or:svy:logistic died irondays sex /*childweigt*/ ///

i.birthinter birthorder multibirth i.timevtCAT i.materage3 i.matereduc ///

i.maritstatus2 contracept i.regioncem ruralurban i.wealth improsani ///

ITNIRS skideli postnatal ANCVISITS ttprotect IPTp surveyyear imprdrkgwat ///

hygincdis delity i.religion2 i.ethnicity cowives hhmem workingcg ///

earlybreastfd watercont NHIStatus hhchu5

*iptp DE

imb surveyyear regioncem wealth matereduc NHIStatus ANCVISITS ,tr(IPTp)

cem surveyyear(#0) regioncem(#0) wealth(#0) ///

matereduc(#0)NHIStatus(#0) ANCVISITS(#0),tr(IPTp) sh

drop if cem_match==0

gen newcemw=new_weight*cem_weights

mi svyset [iweight=newcemw], psu(v021) strata(v022)

mi estimate, esampvaryok or:svy:logistic died IPTp

mi estimate, esampvaryok or:svy:logistic died IPTp sex ///

/*childweigt*/ i.birthinter birthorder multibirth i.timevtCAT ///

materage3 i.matereduc i.maritstatus2 contracept i.regioncem ruralurban ///

i.wealth improsani ITNIRS skideli postnatal ANCVISITS ///

ttprotect /*IPTp*/ irondays surveyyear imprdrkgwat hygincdis delity i.religion2 ///

i.ethnicity cowives hhmem workingcg earlybreastfd watercont NHIStatus hhchu5

*SKILL DE

imb surveyyear regioncem birthorder NHIStatus ANCVISITS multibirth,tr(skideli)

cem surveyyear(#0) regioncem(#0) birthorder(#0) NHIStatus(#0) ANCVISITS(#0) ///

multibirth(#0),tr(skideli) sh

drop if cem_match==0

gen newcemw=new_weight*cem_weights

mi svyset [iweight=newcemw], psu(v021) strata(v022)

mi estimate,or:svy:logistic died skideli

mi estimate, esampvaryok or:svy:logistic died skideli sex ///

childweigt i.birthinter birthorder ///

multibirth i.timevtCAT materage3 i.matereduc i.maritstatus2 contracept ///

i.regioncem ruralurban i.wealth improsani ITNIRS /*skideli*/ postnatal ANCVISITS ///

ttprotect IPTp irondays surveyyear imprdrkgwat hygincdis delity i.religion2 ///

i.ethnicity cowives hhmem workingcg earlybreastfd watercont NHIStatus hhchu5

*Earlybreastfd: EARLY INITIATION OF BREASTFEEDING

imb surveyyear regioncem delity skideli cowives ANCVISITS ,tr(earlybreastfd)

cem surveyyear(#0) regioncem(#0) delity(#0) skideli(#0) ///

cowives(#0) ANCVISITS(#0),tr(earlybreastfd) sh

drop if cem_match==0

gen newcemw=new_weight*cem_weights

mi svyset [iweight=newcemw], psu(v021) strata(v022)

mi estimate, esampvaryok or:svy:logistic died earlybreastfd

mi estimate, esampvaryok or:svy:logistic died earlybreastfd sex ///

childweigt i.birthinter birthorder ///

multibirth i.timevtCAT materage3 i.matereduc i.maritstatus2 contracept ///

i.regioncem ruralurban i.wealth improsani ITNIRS skideli postnatal ANCVISITS ///

ttprotect IPTp irondays surveyyear imprdrkgwat hygincdis delity i.religion2 ///

i.ethnicity cowives hhmem workingcg /*earlybreastfd*/ watercont NHIStatus hhchu5

*ANCVISITS.

imb surveyyear NHIStatus birthinter wealth matereduc ,tr(ANCVISITS)

cem surveyyear(#0) NHIStatus(#0) birthinter(#0) wealth(#0) ///

matereduc(#0) ,tr(ANCVISITS) misets(20) sh

drop if cem_match==0

gen newcemw=new_weight*cem_weights

mi svyset [iweight=newcemw], psu(v021) strata(v022)

mi estimate, esampvaryok or:svy:logistic died ANCVISITS

mi estimate,esampvaryok or:svy:logistic died ANCVISITS sex childweigt ///

i.birthinter birthorder multibirth i.timevtCAT materage3 i.matereduc ///

i.maritstatus2 contracept i.regioncem ruralurban i.wealth improsani ITNIRS ///

skideli postnatal /*ANCVISITS*/ ttprotect IPTp irondays surveyyear imprdrkgwat ///

hygincdis delity i.religion2 i.ethnicity cowives hhmem workingcg earlybreastfd ///

watercont NHIStatus hhchu5

*CLEANPOST DE

imb surveyyear regioncem religion2 ethnicity ANCVISITS, tr(postnatal)

L=0.41

cem surveyyear(#0) regioncem(#0) religion2(#0) ///

ethnicity(#0) ANCVISITS(#0) ,tr(postnatal) misets(20) sh

drop if cem_match==0

gen newcemw=new_weight*cem_weights

mi svyset [iweight=newcemw], psu(v021) strata(v022)

mi estimate, esampvaryok or:svy:logistic died postnatal

mi estimate, esampvaryok or:svy:logistic died postnatal sex childweigt ///

i.birthinter birthorder multibirth i.timevtCAT materage3 ///

i.matereduc i.maritstatus2 contracept i.regioncem ruralurban i.wealth ///

improsani ITNIRS skideli /*postnatal*/ ANCVISITS ttprotect ///

IPTp irondays surveyyear imprdrkgwat hygincdis delity i.religion2 ///

i.ethnicity cowives hhmem workingcg earlybreastfd watercont NHIStatus hhchu5

*hygincdis disposal of stool

imb surveyyear regioncem wealth matereduc birthorder ,tr(hygincdis)

cem surveyyear(#0) regioncem(#0) wealth(#0) ///

matereduc(#0) birthorder(#0) ,tr(hygincdis) misets(20) sh

drop if cem_match==0

gen newcemw=new_weight*cem_weights

mi svyset [iweight=newcemw], psu(v021) strata(v022)

mi estimate, esampvaryok or:svy:logistic died hygincdis

mi estimate,esampvaryok or:svy:logistic died hygincdis sex childweigt ///

i.birthinter birthorder multibirth i.timevtCAT materage3 i.matereduc ///

i.maritstatus2 contracept i.regioncem ruralurban i.wealth improsani ///

ITNIRS skideli postnatal ANCVISITS ttprotect IPTp irondays surveyyear ///

imprdrkgwat /*hygincdis*/ delity i.religion2 ///

i.ethnicity cowives hhmem workingcg earlybreastfd watercont NHIStatus hhchu5

mi test sex

mi test 2.timevtCAT 3.timevtCAT

mi test childweigt

mi test 2.birthinter

mi test birthorder

mi test delity

mi test multibirth

mi test 1.regioncem 2.regioncem

mi test 2.wealth 3.wealth 4.wealth

mi test ruralurban

mi test 1.ethnicity 5.ethnicity 6.ethnicity

mi test 2.religion2 3.religion2 4.religion2

mi test materage3

mi test 1.matereduc 2.matereduc

mi test 1.maritstatus2

mi test cowives

mi test hhchu5

mi test NHIS

*#############################################################################

mi test ANCVISITS

mi test irondays

mi test 1.irondays33

mi test IPTp

mi test ttprotect

mi test skideli

mi test earlybreastfd

mi test hygincdisp

mi test ITNIRS

mi test tiwatsor

mi test watercont

mi test improsani

mi test ITNIRS

mi test contracept

mi test ANC

mi test hygincdisp

mi test postnatal
