# Supplementary material for: Are maternal and child health initiatives helping to reduce under-five mortality in Ghana? Results of a quasi-experimental study using coarsened exact matching
Source: BMC Pediatr. 2021 Oct 25;21:473. doi: 10.1186/s12887-021-02934-3 (PMC8547109; doi:10.1186/s12887-021-02934-3)
Supplement: Supplementary file 2 — Additional file 2. [file 12887_2021_2934_MOESM2_ESM.docx]

Table 1:Before and after matching sample size and imbalance by intervention among children 0-59 months

| **Before matching** | | | | | | | | **After matching** | | | | | | |
| --- | --- | --- | --- | --- | --- | --- | --- | --- | --- | --- | --- | --- | --- | --- |
| **Early initiation of breastfeeding** | | | | | | | | | | | | | | |
| **Sample size** | Unexposed =11,111  Exposed=12,467 | | | | | | | Unexposed=11,066  Exposed=12,467 | | | | | | |
| **Summary of imbalance** | | | | | | | | | | | | | | |
| Overall imbalance | L1=0.19 | | | | | | | L1=2.50 x10^-14^ | | | | | | |
| Pre-treatment covariates | L1 | Mean | Min | 25% | 50% | 75% | Max | L1 | Mean | Min | 25% | 50% | 75% | Max |
| Survey year | 0.00162 | 0.00162 | 0 | 0 | 0 | 0 | 0 | 4.1 x10^-14^ | 6.5x10^-14^ | 0 | 0 | 0 | 0 | 0 |
| Region | 0.04241 | -0.07083 | 0 | -1 | 0 | 0 | 0 | 2.8 x10^-14^ | 4.8 x10^-14^ | 0 | 0 | 0 | 0 | 0 |
| Type of delivery | 0.08244 | -0.08244 | 0 | 0 | 0 | 0 | 0 | 2.5 x10^-15^ | 2.6 x10^-15^ | 0 | 0 | 0 | 0 | 0 |
| Skilled delivery | 0.05376 | 0.05376 | 0 | 0 | 0 | 0 | 0 | 4.4 x10^-14^ | 7.5 x10^-14^ | 0 | 0 | 0 | 0 | 0 |
| Polygamous home | 0.02095 | -0.02095 | 0 | 0 | 0 | 0 | 0 | 3.2 x10^-14^ | 9.2x10^-15^ | 0 | 0 | 0 | 0 | - |
| Antenatal care visits | 0.02844 | 0.02844 | 0 | 0 | 0 | 0 | 0 | 3.0 x10^-14^ | 5.1x10^-14^ | 0 | 0 | 0 | 0 | 0 |
| **Clean postnatal care** | | | | | | | | | | | | | | |
| **Sample size** | Unexposed=19,445  Exposed=4,133 | | | | | | | Unexposed=18,256  Exposed=4,132 | | | | | | |
| **Summary of imbalance** | | | | | | | | | | | | | | |
| Overall imbalance | L1=0.40 | | | | | | | L1=1.77 x10^-14^ | | | | | | |
| Pre-treatment covariates | L1 | Mean | Min | 25% | 50% | 75% | Max | L1 | Mean | Min | 25% | 50% | 75% | Max |
| Survey year | 0.23675 | 0.23675 | 0 | 1 | 0 | 0 | 0 | 6.6 x10^-15^ | -7.5 x10^-15^ | 0 | 0 | 0 | 0 | 0 |
| Region | 0.15833 | -0.15151 | 0 | -1 | 0 | 0 | 0 | 1.2 x10^-14^ | -3.0 x10^-14^ | 0 | 0 | 0 | 0 | 0 |
| Religion | 0.08431 | 0.09842 | 0 | 1 | 0 | 1 | 0 | 6.5 x10^-15^ | -2.2 x10^-14^ | 0 | 0 | 0 | 0 | 0 |
| Ethnicity | 0.16137 | 0.41236 | 0 | 0 | 0 | 0 | 0 | 1.5 x10^-14^ | -9.1 x10^-14^ | 0 | 0 | 0 | 0 | 0 |
| Antenatal care visits | 0.10669 | 0.10669 | 0 | 0 | 0 | 0 | 0 | 8.5 x10^-15^ | 1.1 x10^-14^ | 0 | 0 | 0 | 0 | 0 |
| **Hygienic disposal of stool** | | | | | | | | | | | | | | |
| **Sample size** | Unexposed=14,026  Exposed=9,552 | | | | | | | Unexposed=13,919  Exposed=9,544 | | | | | | |
| **Summary of imbalance** | | | | | | | | | | | | | | |
| Overall imbalance | L1=0.34 | | | | | | | L1=5.57x10^-15^ | | | | | | |
| Pre-treatment covariates | L1 | Mean | Min | 25% | 50% | 75% | Max | L1 | Mean | Min | 25% | 50% | 75% | Max |
| Survey year | 0.12018 | -0.12018 | 0 | 0 | 0 | 0 | 0 | 6.5 x10^-15^ | 1.0 x10^-14^ | 0 | 0 | 0 | 0 | 0 |
| Region | 0.19788 | 0.32124 | 0 | 1 | 0 | 0 | 0 | 5.2 x10^-15^ | -2.6 x10^-15^ | 0 | 0 | 0 | 0 | 0 |
| Wealth | 0.15875 | 0.47174 | 0 | 1 | 1 | 1 | 0 | 1.4 x10^-14^ | -7.1 x10^-15^ | 0 | 0 | 0 | 0 | 0 |
| Maternal education | 0.12856 | 0.23971 | 0 | 1 | 1 | 0 | 0 | 1.4 x10^-14^ | -7.3 x10^-15^ | 0 | 0 | 0 | 0 | 0 |
| Birth order | 0.02097 | -0.02097 | 0 | 0 | 0 | 0 | 0 | 3.3 x10^-15^ | -5.4 x10^-15^ | 0 | 0 | 0 | 0 | 0 |
| **Iron intake** | | | | | | | | | | | | | | |
| **Sample size** | Unexposed=12,231  Exposed=11,347 | | | | | | | Unexposed=12,121  Exposed=11,324 | | | | | | |
| **Summary of imbalance** | | | | | | | | | | | | | | |
| Overall imbalance | L1=0.34 | | | | | | | L1=1.07 x10^-14^ | | | | | | |
| Pre-treatment covariates | L1 | Mean | Min | 25% | 50% | 75% | Max | L1 | Mean | Min | 25% | 50% | 75% | Max |
| Survey year | 0.10953 | 0.10953 | 0 | 0 | 0 | 0 | 0 | 3.4 x10^-15^ | -4.4 x10^-16^ | 0 | 0 | 0 | 0 | 0 |
| Region | 0.08075 | 0.09709 | 0 | 1 | 0 | 0 | 0 | 1.9 x10^-14^ | 6.1 x10^-14^ | 0 | 0 | 0 | 0 | 0 |
| Wealth | 0.12794 | 0.44784 | 0 | 1 | 1 | 1 | 0 | 1.4 x10^-14^ | -2.0 x10^-14^ | 0 | 0 | 0 | 0 | 0 |
| Birth interval | 0.00866 | -0.00866 | 0 | 0 | 0 | 0 | 0 | 1.2 x10^-15^ | 1.3 x10^-15^ | 0 | 0 | 0 | 0 | .- |
| Antenatal care visits | 0.28164 | 0.28164 | 0 | 1 | 0 | 0 | 0 | 1.6 x10^-14^ | 3.0 x10^-14^ | 0 | 0 | 0 | 0 | 0 |
| **Antenatal care visits** | | | | | | | | | | | | | | |
| **Sample size** | Unexposed=4733  Exposed=18,845 | | | | | | | Unexposed=4,730  Exposed=18,476 | | | | | | |
| **Summary of imbalance** | | | | | | | | | | | | | | |
| Overall imbalance | L1=0.36 | | | | | | | L1=9.08x10^-15^ | | | | | | |
| Pre-treatment covariates | L1 | Mean | Min | 25% | 50% | 75% | Max | L1 | Mean | Min | 25% | 50% | 75% | Max |
| Survey year | 0.13881 | 0.13881 | 0 | 0 | 0 | 0 | 0 | 2.1 x10^-15^ | 0.0 | 0 | 0 | 0 | 0 | 0 |
| NHIS | 0.17744 | 0.17744 | 0 | 0 | 1 | 0 | 0 | 4.9 x10^-15^ | 6.9 x10^-15^ | 0 | 0 | 0 | 0 | 0 |
| Birth interval | 0.03666 | -0.03666 | 0 | 0 | 0 | 0 | 0 | 2.3 x10^-15^ | 9.4 x10^-16^ | 0 | 0 | 0 | 0 | 0 |
| Wealth | 0.228 | 0.74224 | 0 | 1 | 1 | 1 | 0 | 1.0x10^-14^ | 1.5 x10^-14^ | 0 | 0 | 0 | 0 | 0 |
| Maternal education | 0.24472 | 0.41287 | 0 | 1 | 1 | 0 | 0 | 1.0 x10^-14^ | 1.4 x10^-15^ | 0 | 0 | 0 | 0 | 0 |
| **Skilled delivery** | | | | | | | | | | | | | | |
| **Sample size** | Unexposed=6,949  Exposed=16,629 | | | | | | | Unexposed=6,921  Exposed=16,299 | | | | | | |
| **Summary of imbalance** | | | | | | | | | | | | | | |
| Overall imbalance | L1=0.42 | | | | | | | L1=1.57 x10^-15^ | | | | | | |
| Pre-treatment covariates | L1 | Mean | Min | 25% | 50% | 75% | Max | L1 | Mean | Min | 25% | 50% | 75% | Max |
| Survey year | 0.15648 | 0.15642 | 0 | 0 | 0 | 0 | 0 | 3.4 x10^-15^ | 5.2 x10^-15^ | 0 | 0 | 0 | 0 | 0 |
| Region | 0.19871 | 0.26338 | 0 | 1 | 0 | 0 | 0 | 6.1 x10^-15^ | 3.2 x10^-14^ | 0 | 0 | 0 | 0 | 0 |
| Birth order | 0.21196 | -0.21196 | 0 | 0 | 0 | -1 | 0 | 6.0 x10^-15^ | 1.3 x10^-15^ | 0 | 0 | 0 | 0 | 0 |
| NHIS | 0.1928 | 0.1928 | 0 | 0 | 1 | 0 | 0 | 7.5 x10^-15^ | 7.5 x10^-15^ | 0 | 0 | 0 | 0 | 0 |
| Antenatal care visits | 0.28138 | 0.28138 | 0 | 1 | 0 | 0 | 0 | 8.8 x10^-16^ | 1.7 x10^-15^ | 0 | 0 | 0 | 0 | 0 |
| Multiple birth | 0.01721 | 0.01721 | 0 | 0 | 0 | 0 | 0 | 3.9 x10^-16^ | 5.2 x10^-18^ | 0 | 0 | 0 | 0 | 0 |
| **Tetanus toxoid vaccine** | | | | | | | | | | | | | | |
| **Sample size** | Unexposed=6,783  Exposed=16,795 | | | | | | | Unexposed=6,564  Exposed=16,456 | | | | | | |
| **Summary of imbalance** | | | | | | | | | | | | | | |
| Overall imbalance | L1=0.36 | | | | | | | L1=8.69x10^-15^ | | | | | | |
| Pre-treatment covariates | L1 | Mean | Min | 25% | 50% | 75% | Max | L1 | Mean | Min | 25% | 50% | 75% | Max |
| Survey year | 0.10031 | 0.10031 | 0 | 0 | 0 | 0 | 0 | 5.9 x10^-15^ | -6.0 x10^-15^ | 0 | 0 | 0 | 0 | 0 |
| Wealth | 0.12078 | 0.39223 | 0 | 1 | 1 | 1 | 0 | 7.4 x10^-14^ | -4.2 x10^-14^ | 0 | 0 | 0 | 0 | 0 |
| Rural/urban residence | 0.06607 | -0.06607 | 0 | 0 | 0 | 0 | 0 | 7.0 x10^-14^ | -7.3 x10^-14^ | 0 | 0 | 0 | 0 | 0 |
| Maternal education | 0.11253 | 0.17621 | 0 | 1 | 1 | 0 | 0 | 8.2 x10^-15^ | -1.5 x10^-15^ | 0 | 0 | 0 | 0 | 0 |
| NHIS status | 0.06553 | 0.0655 | 0 | 0 | 1 | 0 | 0 | 6.1x10^-15^ | -1.5x10^-15^ | 0 | 0 | 0 | 0 | 0 |
| Antenatal care visits | 0.24223 | 0.24223 | 0 | 1 | 0 | 0 | 0 | 4.2 x10^-15^ | -6.6 x10^-15^ | 0 | 0 | 0 | 0 | 0 |
| **Intermittent preventive treatment of malaria in pregnancy (IPT-p)** | | | | | | | | | | | | | | |
| **Sample size** | Unexposed=9,840  Exposed=13,738 | | | | | | | Unexposed=9,435  Exposed=13,550 | | | | | | |
| **Summary of imbalance** | | | | | | | | | | | | | | |
| **Overall imbalance** | L1=0.46 | | | | | | | L1=2.00 x10^-15^ | | | | | | |
| Pre-treatment covariates | L1 | Mean | Min | 25% | 50% | 75% | Max | L1 | Mean | Min | 25% | 50% | 75% | Max |
| Survey year | 0.24622 | 0.24622 | 0 | 1 | 0 | 0 | 0 | 2.8 x10^-15^ | 4.1 x10^-15^ | 0 | 0 | 0 | 0 | 0 |
| Region | 0.0663 | 0.06868 | 0 | 1 | 0 | 0 | 0 | 1.9 x10^-15^ | -8.7 x10^-15^ | 0 | 0 | 0 | 0 | 0 |
| Wealth | 0.0764 | 0.14731 | 0 | 1 | 1 | 0 | 0 | 3.2 x10^-15^ | 3.4 x10^-14^ | 0 | 0 | 0 | 0 | 0 |
| Maternal education | 0.0809 | 0.15081 | 0 | 1 | 1 | 0 | 0 | 4.8 x10^-15^ | 1.6 x10^-14^ | 0 | 0 | 0 | 0 | 0 |
| NHIS status | 0.13536 | 0.13536 | 0 | 0 | 1 | 0 | 0 | 2.8 x10^-15^ | 4.8x10^-15^ | 0 | 0 | 0 | 0 | 0 |
| Antenatal care visits | 0.23336 | 0.23336 | 0 | 1 | 0 | 0 | 0 | 8.0 x10^-15^ | 1.4 x10^-14^ | 0 | 0 | 0 | 0 | 0 |

Note: L1=0 means perfect covariate balance between treated and untreated groups. L1=1 means complete incompatible treatment and comparison groups with respect of covariates. The closer the values of L1 to zero, the better the covariate balance.
