# Supplementary material for: Are maternal and child health initiatives helping to reduce under-five mortality in Ghana? Results of a quasi-experimental study using coarsened exact matching
Source: BMC Pediatr. 2021 Oct 25;21:473. doi: 10.1186/s12887-021-02934-3 (PMC8547109; doi:10.1186/s12887-021-02934-3)
Supplement: Supplementary file 3 — Additional file 3. [file 12887_2021_2934_MOESM3_ESM.docx]

Table 1: Missing data summary

|  | Year | 2008 | | 2014 | | Combined (2008 and 2014) | |
| --- | --- | --- | --- | --- | --- | --- | --- |
|  | Variable | Missing values | Total number of observations | Missing values | Total number of observations | Missing values | Total number of observations |
| 1 | Child’s weight | 16 | 2,029 | 3 | 4,050 | 19 | 6,079 |
| 2 | Birth interval | 5 | 2,040 | 11 | 4,042 | 16 | 6,082 |
| 3 | Delivery type | 2 | 2,043 | 0 | 4,053 | 2 | 6,096 |
| 4 | NHIS status | 0 | 2,045 | 1 | 4,052 | 1 | 6,097 |
| 5 | Polygamous home | 231 | 1,814 | 601 | 3,452 | 832 | 5,266 |
| 6 | Mother’s employment status | 11 | 2,034 | 2 | 4,051 | 13 | 6,085 |
| 7 | Milk given for three days | 28 | 2,017 | 30 | 4,023 | 58 | 6,040 |
| 8 | Mother’s height | 27 | 2,018 | 1,981 | 2,072 | 2008 | 4,090 |
| 9 | Maternal anaemia | 53 | 1,992 | 2,008 | 2,045 | 2061 | 4,037 |
| 10 | Maternal body mass index | 316 | 1,729 | 2,285 | 1,768 | 2601 | 3,497 |

Forty-eight percent (48%) of observations had missing data for at least one variable for the variables numbered 1-10. Only 14% of observations had missing data for at least one variable for the variables numbered 1-6.
