# Supplementary material for: Are maternal and child health initiatives helping to reduce under-five mortality in Ghana? Results of a quasi-experimental study using coarsened exact matching
Source: BMC Pediatr. 2021 Oct 25;21:473. doi: 10.1186/s12887-021-02934-3 (PMC8547109; doi:10.1186/s12887-021-02934-3)
Supplement: Supplementary file 4 — Additional file 4. [file 12887_2021_2934_MOESM4_ESM.docx]

Directed acyclic graphs for each of the eight (8) interventions

Early initiation of breastfeeding

Mortality

Morbidity

Multiple birth

Maternal age

Delivery type

Rural

/urban residence

Maternal

education

Wealth

Religion

Antenatal care visit

Skilled delivery

Ethnicity

NHIS status

**Figure 1: Directed acyclic graph for early initiation of breastfeeding**

Mortality

Morbidity

Clean postnatal care

Maternal education

Rural

/urban residence

Antenatal care visit

Wealth

Religion

Skilled delivery

NHIS status

Ethnicity

**Figure 2: Directed acyclic graph for clean postnatal care**

Hygienic disposal of stool

Mortality

Morbidity

Birth interval

Rural/

urban residence

Region

Wealth

Maternal

education

NHIS status

Antenatal care visit

Religion

Ethnicity

**Figure 3: Directed acyclic graph for hygienic disposal of stool**

Mortality

Morbidity

Skilled delivery

Multiple birth

Maternal age

Rural

/urban residence

Religion

Wealth

Maternal

education

Ethnicity

Antenatal care visit

NHIS status

**Figure 4: Directed acyclic graph for skilled delivery**

Morbidity

Tetanus toxoid vaccine

Mortality

Rural

/urban residence

Maternal education

Religion

Wealth

Ethnicity

Antenatal care visit

NHIS status

**Figure 5: Directed acyclic graph for tetanus toxoid vaccine**

Birthweight

Intermittent preventive treatment of malaria in pregnancy

Morbidity

Mortality

Rural/urban residence

Maternal

education

Wealth

Religion

Antenatal

care visit

NHIS status

Ethnicity

**Figure 6: Directed acyclic graph for intermittent preventive treatment of malaria in pregnancy (IPTp)**

Antenatal care visit

Mortality

Morbidity

Maternal education

Rural

/urban residence

Religion

Wealth

Ethnicity

NHIS status

**Figure 7: Directed acyclic graph for antenatal care visit**

Iron intake for 90 plus+ days

Birth weight

Morbidity

Mortality

Rural

/urban residence

Maternal education

Ethnicity

Wealth

Religion

Antenatal care visit

NHIS status

**Figure 8: Directed acyclic graph for iron intake for 90 plus+ days**
